# Fibroblast Growth Factor 19 Improves LPS-Induced Lipid Disorder and Organ Injury by Regulating Metabolomic Characteristics in Mice

**DOI:** 10.1155/2022/9673512

**Published:** 2022-07-06

**Authors:** Tiantian Liu, Xiaomeng Tang, Yun Cui, Xi Xiong, Yaya Xu, Shaohua Hu, Shuyun Feng, Lujing Shao, Yuqian Ren, Huijie Miao, Hong Zhang, Xiaodong Zhu, Yucai Zhang, Chunxia Wang

**Affiliations:** ^1^Department of Critical Care Medicine, Shanghai Children's Hospital, Shanghai Jiao Tong University School of Medicine, 200062 Shanghai, China; ^2^Institute of Pediatric Infection, Immunity, and Critical Care Medicine, Shanghai Jiao Tong University School of Medicine, 200062 Shanghai, China; ^3^Institute of Pediatric Critical Care, Shanghai Jiao Tong University, 200062 Shanghai, China; ^4^Department of Pediatric Critical Care Medicine, Xinhua Hospital, Shanghai Jiao Tong University School of Medicine, 200092 Shanghai, China; ^5^Department of Clinical Laboratory, Shanghai Children's Hospital, Shanghai Jiao Tong University School of Medicine, 200062 Shanghai, China; ^6^Clinical Research Unit, Shanghai Children's Hospital, Shanghai Jiao Tong University School of Medicine, 200062 Shanghai, China

## Abstract

Sepsis is extremely heterogeneous pathology characterized by complex metabolic changes. Fibroblast growth factor 19 (FGF19) is a well-known intestine-derived inhibitor of bile acid biosynthesis. However, it is largely unknown about the roles of FGF19 in improving sepsis-associated metabolic disorder and organ injury. In the present study, mice were intravenously injected recombinant human FGF19 daily for 7 days followed by lipopolysaccharide (LPS) administration. At 24 hours after LPS stimuli, sera were collected for metabolomic analysis. Ingenuity pathway analysis (IPA) network based on differential metabolites (DMs) was conducted. Here, metabolomic analysis revealed that FGF19 pretreatment reversed the increase of LPS-induced fatty acids. IPA network indicated that altered linoleic acid (LA) and gamma-linolenic acid (GLA) were involved in the regulation of oxidative stress and mitochondrial function and were closely related to reactive oxygen species (ROS) generation. Further investigation proved that FGF19 pretreatment decreased serum malondialdehyde (MDA) levels and increased serum catalase (CAT) levels. In livers, FGF19 suppressed the expression of inducible NO synthase (iNOS) and enhanced the expression of nuclear factor erythroid 2-related factor 2 (NRF2) and hemeoxygenase-1 (HO-1). Finally, FGF19 pretreatment protected mice against LPS-induced liver, ileum, and kidney injury. Taken together, FGF19 alleviates LPS-induced organ injury associated with improved serum LA and GLA levels and oxidative stress, suggesting that FGF19 might be a promising target for metabolic therapy for sepsis.

## 1. Introduction

Sepsis is defined as life-threatening organ dysfunction caused by a dysregulated host response to infection [1]. Sepsis-induced dysregulated lipid metabolism is characterized by decreased serum high-density lipoprotein, total cholesterol, and low-density lipoprotein and elevated triacylglycerols [2]. As the main site for lipid metabolism, the liver suffers from excess free fatty acids (FFAs) resulting in lipotoxicity and sepsis-associated liver injury (SALI) [3]. Therefore, we speculated that improving the lipid metabolic disorder could be a novel therapeutic strategy for SALI.

Fibroblast growth factor 19 (FGF19), as an intestine-derived hormone, enters the liver through the portal vein and suppresses bile acid (BA) and FA synthesis in hepatocytes [4–6]. Given its roles in metabolic regulation, FGF19 is a potential molecular target for type 2 diabetes and nonalcoholic fatty liver disease [7, 8]. Recently, FGF19 analogues or mimics are entering phase 3 clinical research in nonalcoholic steatohepatitis (NASH) [9]. Mechanically, FGF19 binds to FGF receptor 4 (FGFR4) to inhibit cholesterol 7*α*-hydroxylase activity resulting in BA synthesis suppression, and mammalian target of rapamycin complex 1 (mTORC1) is an essential mediator of FGF19 involved in metabolic effects [10, 11]. Moreover, FGF19 promotes FA oxidation and improves mitochondrial dysfunction in skeletal muscle partially through AMP-activated protein kinase (AMPK) signaling pathway [12, 13]. Importantly, recent study proved that lipopolysaccharide (LPS) inhibited the expression of FGFR4 in the livers of mice [14], and our previous study elucidated the association between serum FGF19 level and sepsis-associated gastrointestinal dysfunction [15]. Therefore, we speculated that FGF19 could be a key regulator of metabolism homeostasis and plays a protective role in sepsis-associated metabolic disorder and organ injury.

**Figure 1:**
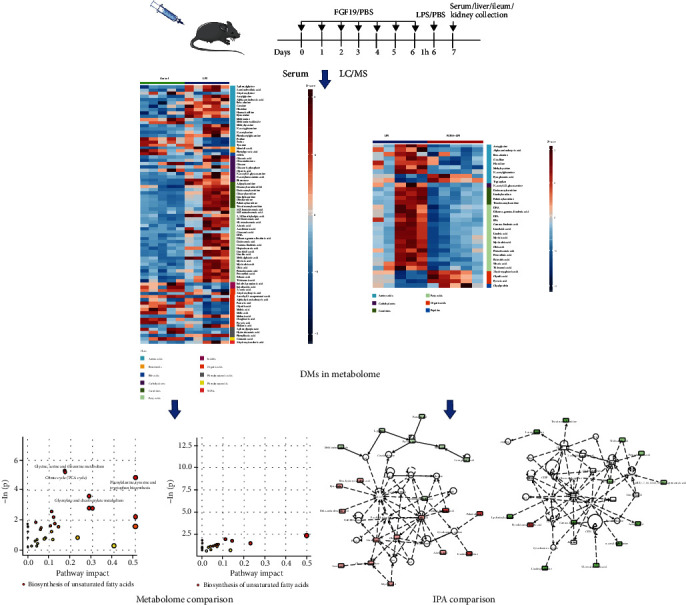
Flowchart illustrating the experimental design of this study.

Metabolomics has been paid more attention in critically ill due to its comprehensive analysis for metabolic alteration [16]. Serum or plasma is usually used for metabolic profile analysis to identify metabolic biomarkers [17–19]. Besides, machine learning method based on integrated pathway analysis (IPA) generating metabolites-related regulatory networks provides a more effective and intuitive way to acquire potential pathological mechanisms [20–22]. In the present study, we aimed to explore the role of FGF19 in LPS-induced serum metabolic profile disturbance in mice using targeted liquid chromatography coupled with mass spectrometry (LC-MS) (Figure 1).

## 2. Materials and Methods

### 2.1. Animal

A total of 48 male C57BL/6 J mice (8-10 weeks old) were purchased from Shanghai Southern Model Animal Experimental Center (Shanghai, China). Mice were caged on a 12-h light/dark cycle at 25°C and were allowed free access to tap water and a standard rodent chow ad libitum. Mice were randomly divided into four groups including control, LPS, only FGF19 treatment (FGF19), and FGF19 pretreatment followed by LPS administration (FGF19 + LPS). To construct endotoxemia model to mimic sepsis and sepsis-associated organ dysfunction, mice were intraperitoneally injected with LPS (5 mg/kg, E. coli 0111: B4, Sigma-Aldrich Co.) for 24 h [23]. Recombinant human FGF19 (Novoprotein, China) was intravenously injected *via* tail vein at a dose of 0.1 mg/kg body weight daily for 7 days before LPS treatment. The management and experimental care of mice were conducted in accordance with the protocol that was approved by the Ethics Committee of Shanghai Children's Hospital, Shanghai Jiao Tong University School of Medicine (Shanghai, China).

Mice were sacrificed at 24 h after LPS administration. Blood samples were collected and centrifuged at 3000 rpm for 15 min to separate serum. The serum samples from a batch of animals (6 mice per group) were sent for metabolomic experiments. The liver, ileum, kidney tissues, and serum samples of the other batch of animals (6 mice per group) were collected and performed to molecular biological experiments. In addition, liver tissues were flash frozen in liquid nitrogen for further analysis.

### 2.2. Sample Preparation and LC/MS Analysis

Metabolomic analysis was conducted using Q300 Kit (Metabo-Profile Biotechnology, Shanghai, China). Due to quality control (QC) test, 20 QC-passed serum samples (5 mice per group) were further analyzed by targeted metabolomics. Detailed methods and statistical analysis for metabolomic data are available in the Supplementary Materials (available here).

### 2.3. Integrated Analysis of Metabolomics

IPA software (IPA China, provided by Shanghai Jiao Tong University School of Medicine) was used to analyze differential metabolites (DMs) based on metabolomics to construct molecular interaction network models (comparison analysis).

### 2.4. Histological Examination

Liver, ileum, and kidney tissues were harvested and fixed in 4% paraformaldehyde overnight and next embedded in paraffin. 4-*μ*m thickness tissue sections were stained with hematoxylin and eosin (H&E), and images were captured randomly under light microscopy. Liver, ileum, and kidney injury scores were determined as described before [24–26]. All injury assessments were performed by two observers who were unaware of the treatment. The final score was expressed as an average grade.

### 2.5. Liver Immunohistochemical (IHC) and Immunofluorescence (IF) Staining

Liver tissue sections were incubated with primary and then secondary antibodies following the routine protocols of IHC and IF. For IHC staining, anti-inducible NO synthase (iNOS) antibody was purchased from Abcam (ab3523, UK). For IF staining, anti-hemeoxygenase-1 (HO-1) antibody was purchased from CST (#43966, USA). Horse radish peroxidase-conjugated or fluorescence-conjugated secondary antibody (Rabbit) was purchased from eBioscience (Shanghai, China).

### 2.6. Biochemical Analysis for Oxidative Stress

Serum levels of catalase (CAT) and malondialdehyde (MDA) were measured using corresponding commercial assay kits according to the manufacturer's instructions (Nanjing Jiancheng Bioengineering Institute, China).

### 2.7. Quantitative Real-Time Polymerase Chain Reaction (RT-qPCR)

The total RNA from liver tissue was prepared with TRIzol reagent (Invitrogen Life Technologies, Carlsbad, CA, USA). qPCR was performed on an ABI 7500 system (Applied Biosystem, Foster, CA, USA). The relative mRNA expressions for targeted genes were normalized by reference gene *Gapdh* and were calculated by 2^−*ΔΔ*CT^ method. The sequences of primers used for RT-qPCR were showed in Supplementary Materials.

### 2.8. Western Blotting

Liver tissues were homogenized in RIPA lysis buffer (Beyotime Biotechnology). The following antibodies were used in this study: anti-*β*-Actin (1: 1000, #3700, CST), anti-Cytochrome c (1 : 1000, #3700, CST), anti-Cleaved Caspase-3 (1 : 1000, #9664, CST), anti-HO-1 (1 : 1000, #10701, Proteintech), anti-NRF2 (1 : 1000, #16396, Proteintech), and the secondary anti-Rabbit antibody (eBioscience, Shanghai, China). Relative protein levels were obtained by normalized to *β*-Actin. The intensities of protein band were analyzed by ImageJ software.

### 2.9. Statistical Analysis

Normally distributed continuous variables are presented as mean ± SEM, and Student *t-*test was used to compare the difference between two groups. GraphPad Prism version 8 (GraphPad Software, La Jolla, CA, USA) was used for comparison analysis. *P* < 0.05 was considered statistically significant.

## 3. Results

### 3.1. Serum Overall Metabolic Profiles in Mice

**Figure 2:**
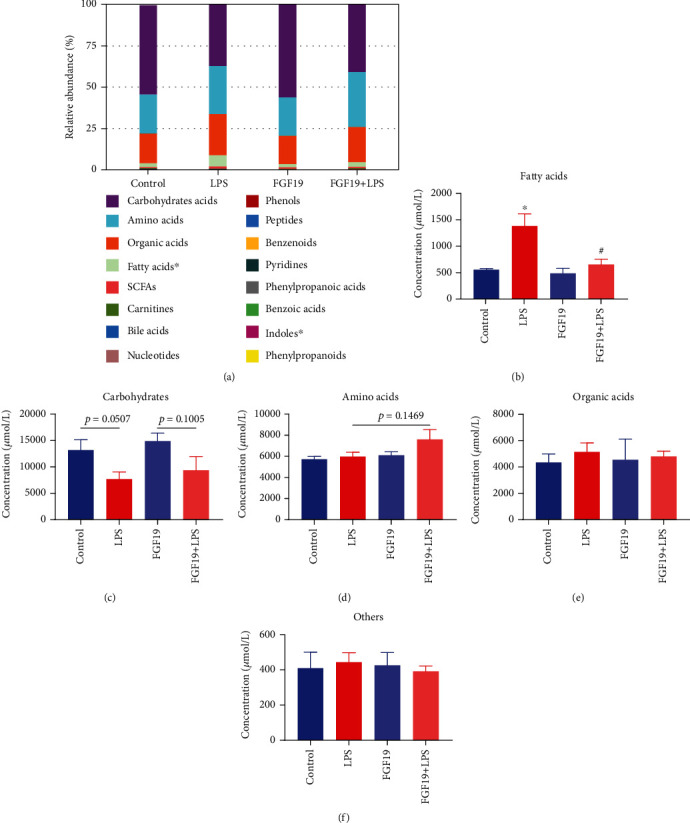
The relative abundance and concentration of 16 metabolite classes in different groups. Mice were divided into four groups including control, LPS, only FGF19 treatment (FGF19), and pretreatment with FGF19 followed by LPS administration (FGF19 + LPS) (*n* = 5). (a) The relative abundance of 16 metabolite classes. The abscissa of stacked bar chart indicates groups, and the ordinate indicates the relative abundance. Different colors indicate different metabolite classes. ^∗^ indicates *P* < 0.05 using analysis of variance (ANOVA). (b–f) The concentration of special metabolite class including fatty acids (b), carbohydrates (c), amino acids (d), organic acids (e), and others (f). ^∗^ indicates *P* < 0.05 for LPS *vs.* control, ^#^ indicates *P* < 0.05 for FGF19 + LPS *vs.* LPS, using Student's *t-*test.

There were 207 DMs in sera among four groups, which were divided into 16 classes, including carbohydrates, amino acids, organic acids, FAs, short-chain fatty acids (SCFAs), carnitines, BAs, nucleotides, phenols, peptides, benzenoids, pyridines, phenylpropanoic acids, benzoic acids, indoles, and phenylpropanoids. Among them, carbohydrates, amino acids, organic acids, and FAs were the major metabolite components. LPS remarkably increased the levels of FAs, and FGF19 reversed LPS-induced effect in mice (Figures 2(a) and 2(b)). Furthermore, FGF19 decreased the mRNA levels of fatty acid synthase (*Fasn*), ATP-citrate lyase (*Acly*), and increased the mRNA levels of fatty acid transport protein 1 (*Fatp1*) and carnitine palmitoyltransferase 1*α* (*Cpt1α*) in the livers in response to LPS (Figure S1). However, there were not significantly differences in serum carbohydrates levels, amino acids levels, organic acids levels, or others in the FGF19 + LPS group compared with LPS group (Figures 2(c)–2(f)).

### 3.2. Multidimensional Statistics Analysis of Serum Metabolic Profile

**Figure 3:**
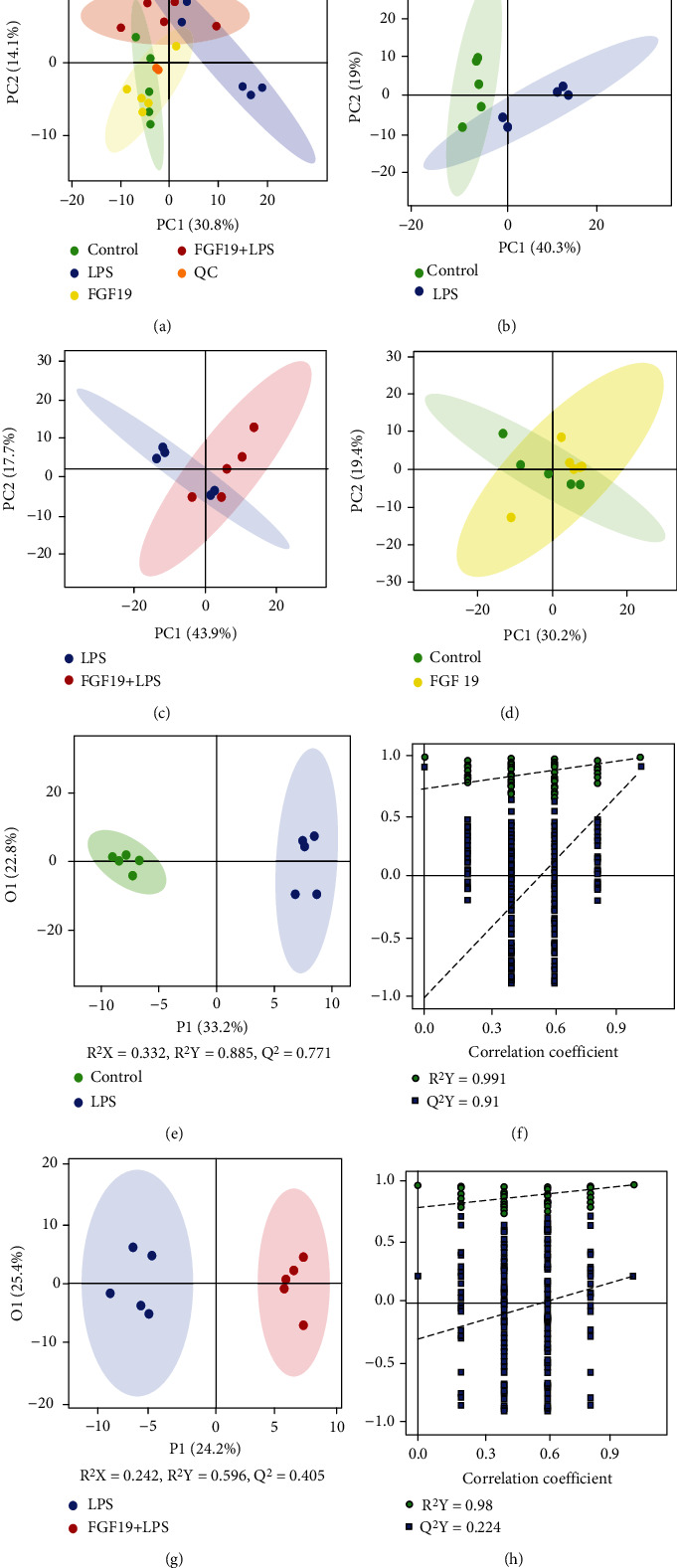
PCA scatter plot and OPLS-DA analysis of metabolite profile. Mice were divided into four groups including control, LPS, only FGF19 treatment (FGF19), and pretreatment with FGF19 followed by LPS administration (FGF19 + LPS) (*n* = 5). (a) Score plot with aggregated quality control (QC) samples, (b–d) 2D PCA plot of LPS *vs.* control group (b), FGF19 + LPS *vs.* LPS group (c), FGF19 *vs.* control group (d). (e–h) OPLS-DA score scatter plots and permutation test of OPLS-DA model of LPS *vs.* control group (e, f) and FGF19 + LPS *vs.* LPS group (g, h). The abscissa indicates the displacement retention of the permutation test, and the ordinate indicates the value of R2Y or Q2Y. The green dot indicates the R2Y value obtained by the displacement test, the blue square indicates the Q2Y value obtained by the permutation test, and the two dotted lines indicate the regression lines of R2Y and Q2, respectively. The point where the displacement retention is 1 is R2Y and Q2 of the original model.

In the PCA model, QC samples were successfully separated from the tested samples and clustered together (Figure 3(a)). The metabolites in sera of mice in the LPS group or FGF19 + LPS group were well separated from the control or LPS group in the 2D PCA plot, respectively (Figures 3(b) and 3(c)). However, there was no significantly separation between FGF19 group and control group (Figure 3(d)). Moreover, OPLS-DA score plots displayed similar results as shown in the 2D PCA plot (Figures 3(e) and 3(g)). Further permutation test proved that the OPLS-DA model have good robustness without overfitting (Figures 3(f) and 3(h)).

### 3.3. Differential Metabolites and Potential Biomarkers Screened from Serum Metabolomics in Mice

**Figure 4:**
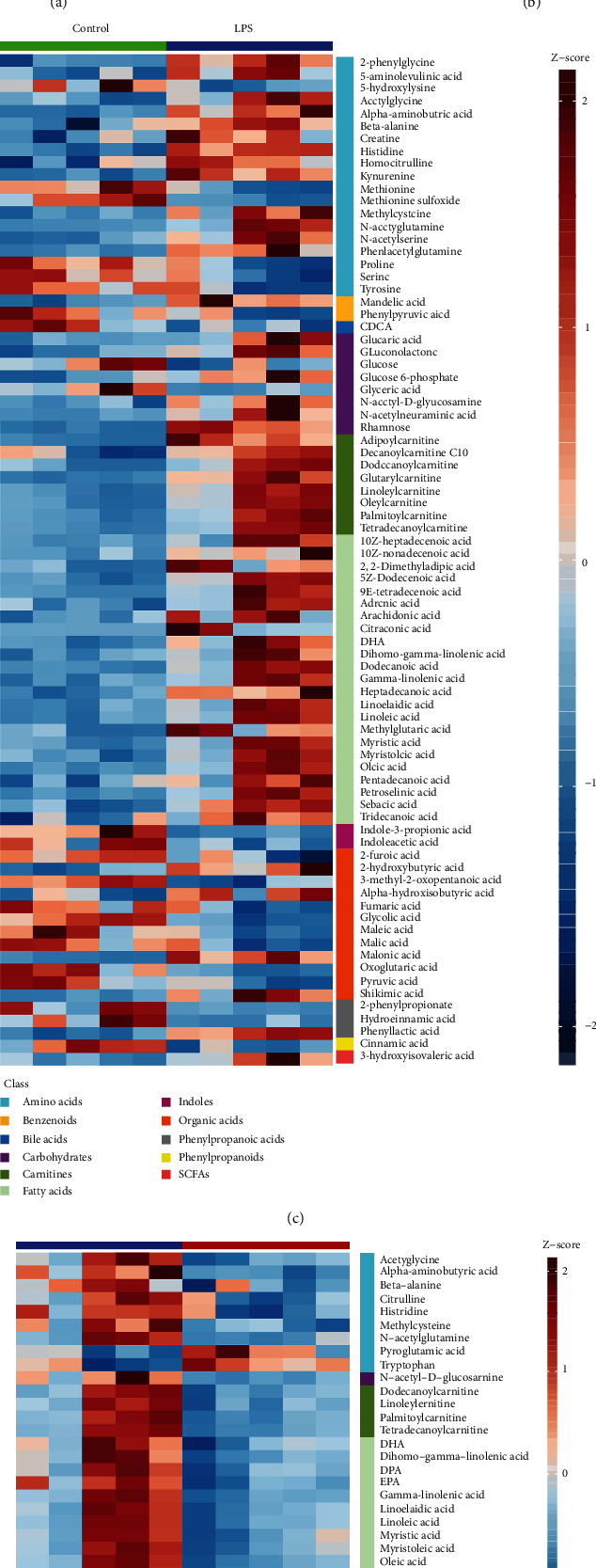
Differential metabolites (DMs) identified in the multivariate OPLS-DA model and univariate statistical analysis. (a, c) LPS *vs.* control; (b, d) FGF19 + LPS *vs.* LPS (*n* = 5). (a, b) Volcano plot representation of DMs. Each point in the map represents one kind of metabolite. Scatter color represents the final screening result, red highlight represents significant upregulation; blue highlight represents significant downregulation, and gray represents nonsignificant DMs. The dataset was screened using VI*P* value >1 and *P* < 0.05. (c, d) Heatmaps of DMs. Each column depicts a sample, and each row represents one kind of metabolite. The color of each part corresponds to the concentration value of each metabolite (red, upregulated; blue, downregulated). (e) Venn diagram of the numbers of DMs of LPS *vs.* control and FGF19 + LPS *vs.* LPS.

According to variable importance in projection (VIP) >1 and *P* < 0.05, based on OPLS-DA multivariate data analysis and univariate statistics, a total of 82 DMs were found in the LPS group compared with control group, including 58 upregulated metabolites and 24 downregulated metabolites (Figure 4(a)). There were 33 DMs in the FGF19 + LPS group compared with LPS group, including 6 upregulated metabolites and 27 downregulated metabolites (Figure 4(b)). There were 25 DMs appeared in both LPS vs. control and FGF19 + LPS vs. LPS group at the same time. Potential metabolites affected by FGF19 in response to LPS included 6 kinds of amino acids (histidine, *β*-alanine, methylcysteine, *α*-aminobutyric acid, acetylglycine, and N-acetylglutamine), 12 kinds of fatty acids (sebacic acid, tridecanoic acid, myristoleic acid, myristic acid, pentadecanoic acid, gamma-linolenic acid, linoleic acid, linoelaidic acid, dihomo-gamma-linolenic acid, DHA, oleic acid, and petroselinic acid), 1 kind of carbohydrate (N-acetyl-D-glucosamine), 4 kinds of carnitines (linoleyl carnitine, dodecanoylcarnitine, palmitoylcarnitine, and tetradecanoylcarnitine), and 2 kinds of organic acids (glycolic acid and pyruvic acid). Among these metabolites, amino acids, FAs, carbohydrate, and carnitines were increased, while organic acids were decreased in response to LPS. Intriguingly, FGF19 almost totally reversed these changed induced by LPS (Figures 4(c)–4(e)).

### 3.4. Metabolic Enrichment and Pathway Analysis of Differential Metabolites

**Figure 5:**
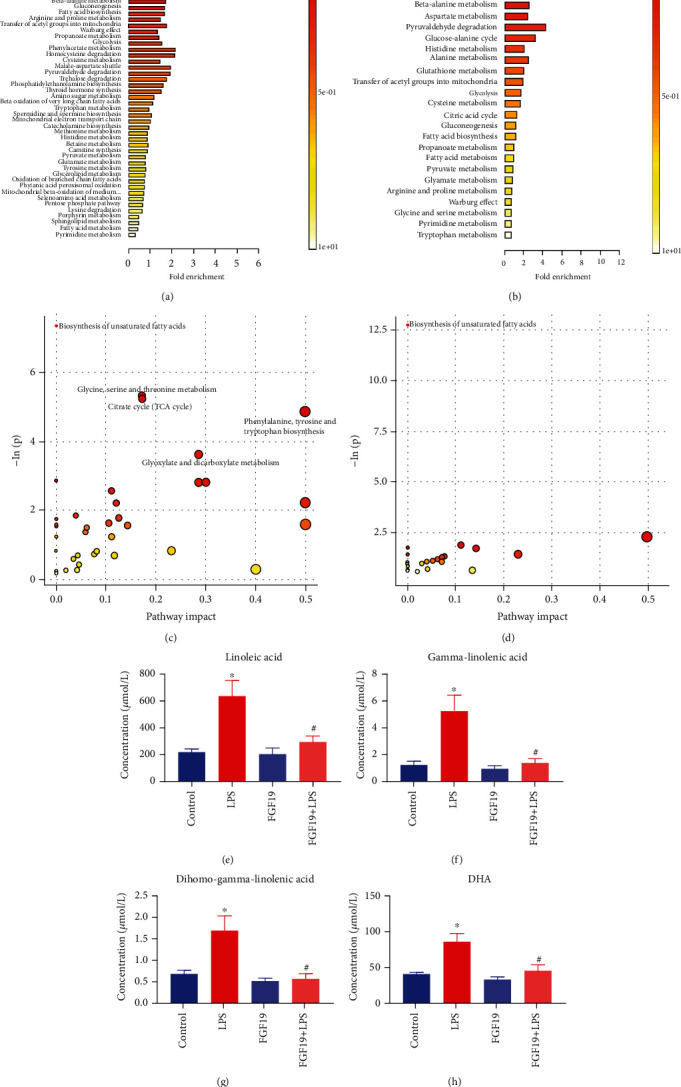
Pathway enrichment analysis using pathway-associated metabolite sets (SMPDB) and pathway analysis bubble plot by mmu set. Mice were divided into four groups including control, LPS, only FGF19 treatment (FGF19), and pretreatment with FGF19 followed by LPS administration (FGF19 + LPS) (*n* = 5). (a, b) Pathway enrichment analysis. Different colors represent different *P* values. The smaller *P* value, the redder the color is. The length of line segment in SMPDB represents fold enrichment. (c, d) Pathway analysis bubble plot by mmu set. The abscissa of pathway analysis bubble plot represents pathway impact, and the ordinate indicates -ln(p). (a, c) LPS *vs.* control, (b, d) FGF19 + LPS *vs.* LPS. The concentration of differentially significant metabolites in common pathway between LPS vs. control and FGF19 + LPS vs. LPS. (e) Linoleic acid, (f) gamma-linolenic acid, (g) dihomo-gamma-linolenic acid, and (h) (DHA). ^∗^ indicates *P* < 0.05 for LPS vs. control, and ^#^ indicates *P* < 0.05 for FGF19+LPS vs. LPS, using Student's t-test. Each black point represents a sample.

Pathway enrichment analysis revealed that *α*-linolenic acid (*α*-LA) and linoleic acid (LA) metabolism were significantly enriched in DMs in either LPS group compared with control group or FGF19 + LPS group compared with LPS group (Figures 5(a) and 5(b)). Detailed pathway analysis indicated that biosynthesis of USFAs was the potential pathway contributed to FGF19-mediated changes of metabolites in response to LPS stimuli (Figures 5(c) and 5(d)). Furthermore, LA, gamma-linolenic acid (GLA), dihomo-gamma linolenic acid (DGLA), and DHA were significantly decreased in sera of FGF19-pretreated mice (Figures 5(e)–5(h)).

### 3.5. Network and Comparison Analysis in Integrated Pathway Analysis

**Figure 6:**
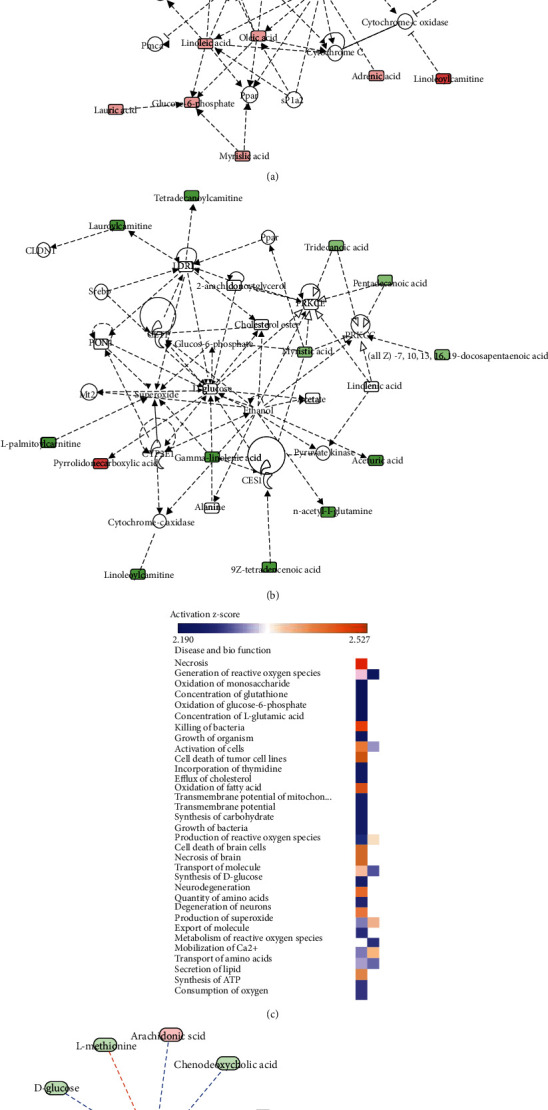
Network function analysis and comparison analysis of differential metabolites (DMs) using IPA software. (a, b) Top-ranked enriched networks based on differential metabolites (DMs). Red, significantly increased; green, significantly decreased. (a) LPS *vs.* control; (b) FGF19 + LPS *vs.* LPS. (c) Disease and biofunction analysis based on comparison analysis (orange, upregulated; blue, downregulated). Left, LPS *vs.* control; right, FGF19 + LPS *vs.* LPS. (d, e) Detailed metabolites related to generation of reactive oxygen species (ROS) in response to LPS. (d) Only LPS treatment. (e) With FGF19 pretreatment (red, significantly increased; green, significantly decreased; orange arrow, activated; blue arrow, inhibited).

The 82 DMs in response to LPS or 33 DMs related to FGF19 pretreatment in response to LPS were submitted to IPA. Network function analysis revealed that 82 DMs were involved in “immunological disease, inflammatory disease, and inflammatory response” with a score of 48, and 33 DMs were mainly responsible for “cellular compromise, lipid metabolism, and small molecule biochemistry” with a score of 33 (Figures 6(a) and 6(b)). Interestingly, we found LA was related to NADPH oxidase (NOS) and cytochrome-c oxidase (COX) in the LPS group, while GLA was relevant to superoxide and COX in the FGF19 + LPS group. Next comparison analysis demonstrated that FGF19 pretreatment inhibited LPS-induced necrosis and reactive oxygen species (ROS) production (Figure 6(c)). Moreover, further analysis based on comparison analysis also enclosed that LA and GLA were involved in generation of ROS (Figures 6(d) and 6(e)).

### 3.6. FGF19 Pretreatment Alleviates LPS-Induced Organ Injury

**Figure 7:**
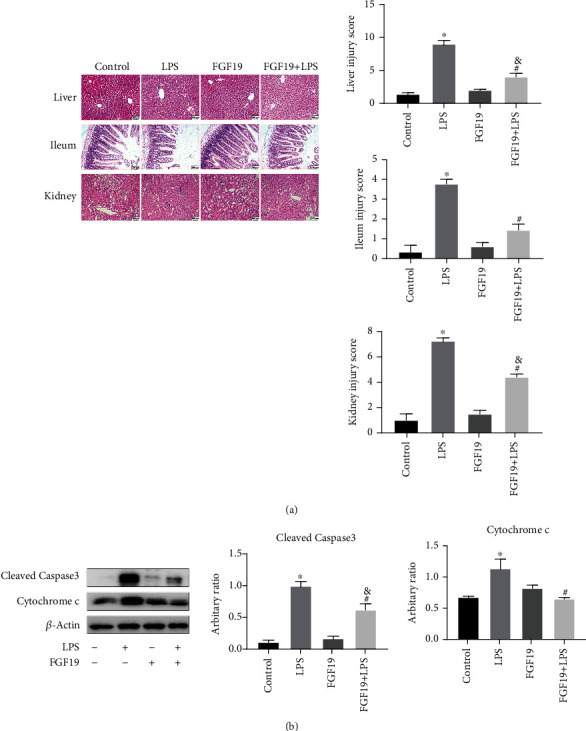
FGF19 alleviates LPS-induced organ injury. Mice were divided into four groups including Control, LPS, only FGF19 treatment (FGF19), and pretreatment with FGF19 followed by LPS administration (FGF19+LPS) (*n* = 6). (a) H&E staining for liver, ileum, and kidney (20 ×) at 24 h after LPS treatment in the indicated groups of mice. Scale bars, 200 *μ*m. Liver, ileum, and kidney injury scores were determined according to the scoring criteria described in the Methods section. (b) Cleaved Caspase-3 and Cytochrome c protein levels in the livers. All data are presented as mean ± SEM. ^∗^ indicates the significant difference compared with control group, ^&^ indicates the significant difference compared with FGF19 group, ^#^ indicates the significant difference compared with LPS group.

Histological analysis indicated that FGF19 pretreatment partially ameliorated LPS-induced liver, ileum, and kidney injury, displaying significantly lower injury scores (Figure 7(a)). Furthermore, FGF19 pretreatment significantly inhibited LPS-induced expression of Cleaved Caspase-3 and Cytochrome c in the liver (Figure 7(b)).

### 3.7. FGF19 Pretreatment Improves LPS-Induced Oxidative Stress

**Figure 8:**
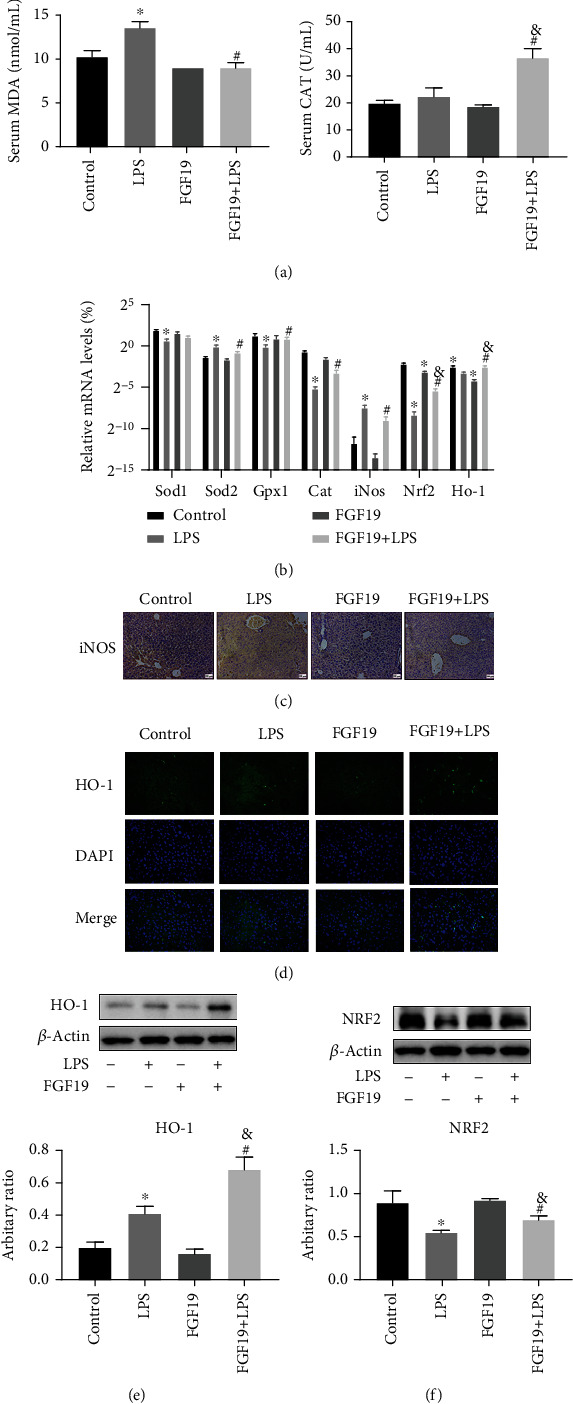
FGF19 improves LPS-induced oxidative stress. Mice were divided into four groups including Control, LPS, only FGF19 treatment (FGF19), and pretreatment with FGF19 followed by LPS administration (FGF19+LPS) (*n* = 6). (a) Serum levels of MDA and CAT. (b) The mRNA level of *Sod1*, *Sod2*, *Gpx1*, *Cat*, *iNos*, *Nrf2*, and *Ho-1* in the livers. (c) Immunohistochemical staining against iNOS in the livers. Scale bars, 200 *μ*m. (d) Immunofluorescent staining against HO-1 (green) in livers and DAPI (blue) was used to highlight the cell nucleus (40×). (e) NRF2 protein levels in the livers. (f) HO-1 protein levels in the livers. All data are presented as mean ± SEM. ^∗^ indicates the significant difference compared with control group, ^&^ indicates the significant difference compared with FGF19 group, ^#^ indicates the significant difference compared with LPS group.

IPA results suggested that FGF19 pretreatment ameliorated LPS-induced organ injury and inflammatory response, which might be related to generation of ROS. Thus far, we run a series of validation tests to verify relationship of FGF19 and oxidative stress. Interestingly, serum MDA level was decreased, and serum CAT level was increased in FGF19-pretreated mice (Figure 8(a)). The mRNA levels of glutathione peroxidase 1 (*Gpx1*) and *Cat* in livers were significantly higher in mice pretreated with FGF19 compared with LPS-treated mice, but not superoxide dismutase (*Sod1* and *Sod2*) (Figure 8(b)). Consistently, both the mRNA and protein levels of iNOS were significantly decreased in the FGF19-pretreated group compared with LPS group (Figures 8(b) and 8(c)). Furthermore, FGF19 pretreatment promoted the LPS-suppressed expression of NRF2 and HO-1 in the livers (Figures 8(b) and 8(d)–8(f)).

## 4. Discussion

**Figure 9:**
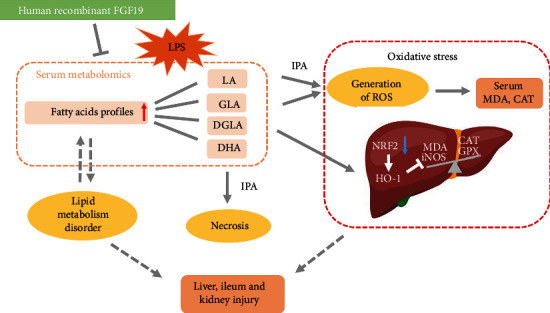
Working model for FGF19-mediated fatty acid profiles involved in LPS-induced organ injury and oxidative stress. IPA: ingenuity pathway analysis; FGF19: fibroblast growth factor 19; LPS: lipopolysaccharide; LA: linoleic acids; GLA: gamma linolenic acid; DGLA: dihomo-gamma linolenic acid; MDA: malondialdehyde; CAT: catalase; GPX: glutathione peroxidase; iNOS: inducible NO synthase; NRF2: nuclear factor erythroid 2-related factor 2; HO-1: hemeoxygenase-1.

Metabolic disturbance is a critical characteristic of sepsis and also is a potential entry for developing novel therapeutic strategy for sepsis. In the present study, we found that FGF19 pretreatment attenuates LPS-induced organ injury associated with LA/GLA-ROS generation-NRF2/HO-1 pathway (Figure 9). To the best of our knowledge, it is the first report about FGF19 regulating serum LA/GLA levels involved in sepsis-associated organ injury, which gives a new sight into the metabolic strategy for sepsis treatment.

Metabolic response to LPS in healthy people is concordant with that of community-acquired sepsis survivor [27]. LPS-induced mice model was reported to be used for investigating the potential metabolites involved in improving organ injury [28]. As described in our previous studies [29, 30], LPS-induced mice model was used in this study. We previously reported that serum FGF19 level was decreased in patients with sepsis-associated gastrointestinal dysfunction [15]. The gut barrier failure leads to increased levels of inflammatory factors and absorption of LPS and decreased bacteria clearance, aggravating SALI [31]. Moreover, FGF19 regulates FA metabolism for treatment of NASH [32]. In this study, we revealed that FGF19 partially suppresses LPS-induced FA synthesis and promotes FA transport and *β*-oxidation in livers and influences FAs levels including oleic acid, LA, and GLA in serum. This study gave a new insight into FGF19-mediated changes of FA profiles in response to LPS. Considering the characteristic of intestine-derived, FGF19 could be a potential therapeutic target for sepsis from the view of FA regulation.

Through analyzing the DMs between LPS *vs*. control group and LPS *vs*. FGF19 + LPS group, 25 DMs were screened and potentially involved in the protective roles of FGF19 in LPS-induced metabolic disorders. Among these, 12 fatty acids were involved. For example, myristic acid is a saturated FA, whose increasement might be related to the alteration of lipid metabolism and energy production in sepsis [33, 34]. In a cohort of septic patients and patients with systemic inflammatory response syndrome (SIRS), serum myristic acid was significantly higher in sepsis and SIRS group, which indicates myristic acid should be considered as a new candidate marker for sepsis [35]. In summary, FGF19 pretreatment mainly improved LPS-induced lipid metabolism, indicating FGF19 might be closely associated with improved metabolic disturbance of sepsis. Aldafermin (NGM282), an FGF19 engineered analogue, is mainly applied in clinical trial including NASH [36] and primary sclerosing cholangitis [37]. Currently, NGM282 is applied in phase 2 clinical trials in NASH showing favorable clinical effect on reducing liver fat and improving fibrosis (ClinicalTrials.gov, Number: NCT02443116) [38]. In atherosclerosis, both FGF19 and NGM282 regulated cholesterol homeostasis and improved hepatic steatosis in *db/db* mice [6]. In the future, the potential therapeutic values of NGM282 or other FGF19 analogue in sepsis should be conducted in a well-designed clinical trial.

Our detailed pathway analysis results showed that FGF19 pretreatment suppressed USFA biosynthesis and LA and GLA were involved. Peritoneal fluid metabolomics or serum metabolomics revealed that LA level was increased in rodent treated by LPS, and biosynthesis of USFA was influenced [39, 40]. In agreement with these previous study, LPS increased LA, GLA, DGLA, and DHA. Instead, FGF19 pretreatment reversed the effect. LA is converted to arachidonic acid (AA) *via* GLA and DGLA, and LA is converted to DHA *via α*-LA [41, 42]. The levels of AA were increased by a two-fold in patients with severe sepsis [43]. Moreover, LPS-induced LA and AA led to increased FFAs and prostaglandin E2 in serum resulting in organ injury [44]. We suspected that LPS-induced LA metabolism disturbance is earlier than organ injury. Accumulated evidences indicated that USFA are substrates for the synthesis of multiple molecules that are active in inflammatory response [45–48]. Decreased Omega 6/Omega 3 FAs were proved to attenuate ethanol-induced liver injury [49]. So, we suspected that FGF19 improves LPS-induced organ injury mainly *via* downregulating LA metabolism. However, the detailed mechanisms underlying FGF19 affecting LA and GLA levels need further investigation in the future. Serum metabolomics could be affected by diet and gut microbiome, and it is limited to analyze metabolites produced endogenously [50]. So, the role of supplement to specific microbial metabolites and analysis of tissue metabolomics need further investigation in the future.

Imbalance of oxygen metabolism was manifested as increased oxygen demand and aerobic anaerobic respiration in a sepsis model in rats [40]. Metabolomic data indicated that FGF19 improved the level of pyruvic acid, suggesting FGF19 possibly improves LPS-induced mitochondria dysfunction. FFAs overload results in incomplete oxidation in the mitochondria, peroxisomes, and microsomes, leading to the generation of ROS [51, 52]. LA could produce more mitochondria-derived ROS than other FFAs [53]. In the present study, IPA network showed that FGF19-induced changes of LA and GLA levels were associated with ROS generation in response to LPS. Otherwise, FGF19 could protect oxidative stress-induced diabetic cardiomyopathy *via* activation of AMPK/NRF2/HO-1 pathway [54]. In our study, FGF19 strongly induced NRF2 and HO-1 expression and suppressed iNOS expression in livers in response to LPS. Moreover, FGF19 increased serum CAT and hepatic *Cat* and *Gpx1* mRNA expression. So, we supposed that FGF19 pretreatment might promote ROS clearance related to activating antioxidant enzymes.

Generally, the serum metabolomic profile through mice intraperitoneal-injected LPS with or without FGF19 pretreatment, IPA analysis, and our further validation tests revealed FGF19 improved LPS-induced lipid metabolic disorder and organ injury, which was associated with LA/GLA-generation of ROS pathway. However, the present study also has several limitations. The sample size for serum metabolomic analysis (*n* = 5) was small. LPS-induced mice model was only model used in this study. Thus, the conclusion should be further assessed in future studies. Moreover, serum LA and GLA levels in patients with sepsis could be paid more attention to monitor routinely, and potential relationship between serum LA and GLA levels and the outcome of sepsis should be further investigated in the future.

## 5. Conclusion

FGF19 pretreatment alleviates LPS-induced metabolic disorder and organ injury. Integrated pathway analysis implied that FGF19-reduced serum LA and GLA levels might be correlated to improving oxidative stress, and further experimental data confirmed that FGF19 activates NRF2/HO-1 pathway in response to LPS. So, FGF19 could be potential metabolic regulator during sepsis and therapeutic target for sepsis treatment.

## Data Availability

The datasets generated and analyzed during the current study are available from the corresponding author on reasonable request.

## Abbreviations

FGF19: Fibroblast growth factor 19
LPS: Lipopolysaccharide
IPA: Ingenuity pathway analysis
DMs: Differentially metabolites
LC-MS: Liquid chromatography coupled with mass spectrometry
LA: Linoleic acids
GLA: Gamma-linolenic acid
DGLA: Dihomo-gamma linolenic acid
ROS: Reactive oxygen species
MDA: Malondialdehyde
CAT: Catalase
iNOS: Inducible NO synthase
NRF2: Nuclear factor erythroid 2-related factor 2
HO-1: Hemeoxygenase-1
PCA: Principal component analysis
OPLS-DA: Orthogonal partial least squares discriminant analysis
VIP: Variable importance in projection
SMPDB: Pathway-associated metabolites sets
FFA: Free fatty acid
BA: Bile acid
SALI: Sepsis-associated liver injury
AMPK: AMP-activated protein kinase
NASH: Nonalcoholic steatohepatitis
FGFR4: FGF receptor 4
Gpx1: Glutathione peroxidase 1
Sod: Superoxide dismutase
COX: Cytochrome-c oxidase
AA: Arachidonic acid
SIRS: Systemic inflammatory response syndrome
*α*-LA: *α*-Linolenic acid.
