# Supplementary material for: Fibroblast Growth Factor 19 Improves LPS-Induced Lipid Disorder and Organ Injury by Regulating Metabolomic Characteristics in Mice
Source: Oxid Med Cell Longev. 2022 Jul 6;2022:9673512. doi: 10.1155/2022/9673512 (PMC9279090; doi:10.1155/2022/9673512)
Supplement: Supplementary Materials — The Supplementary Material for this article can be found online. [file 9673512.f1.zip › Supplementary materials (2).docx]

**Supplementary Materials and Methods**

**Sample preparation and LC/MS analysis**

On the Biomek 4000 workstation (Biomek 4000, Beckman Coulter, Inc., Brea, California, USA), 25 μL of serum and 120 μL of ice-cold methanol with partial internal standards were vortexed vigorously for 5 min. After centrifugation, 30 μL supernatant was used to derivatization with 20 μL of freshly prepared derivative reagents. After derivatization, 135 μL of supernatant centrifugated with 330 μL of ice-cold 50% methanol solution was mixed with 10 μL of internal standards. Subsequently, the derivatized samples and serial dilutions of derivatized stock standards were randomly analyzed and quantitated by an ultraperformance liquid chromatography coupled to tandem mass spectrometry (UPLC-MS/MS) system (ACQUITY UPLC-Xevo TQ-S, WatersCorp., Milford, MA, USA) to quantitate all targeted metabolites. The standards of targeted metabolites were obtained from Sigma-Aldrich (St. Louis, MO, USA), Steraloids Inc. (Newport, RI, USA) and TRC Chemicals (Toronto, ON, Canada).

**Statistical Analysis for Metabonomic Data**

For data processing, the raw data files generated by UPLC-MS/MS were processed using MassLynx software (v4.1, Waters, Milford, MA, USA) to perform peak integration, calibration, and quantitation for each metabolite. Principal component analysis (PCA), and orthogonal partial least squares discriminant analysis (OPLS-DA) were also performed. Variable importance in projection (VIP) was obtained based on the OPLS-DA model. Metabolites with VIP > 1 and *P* < 0.05 (univariate analyses were based on whether the data were normally distributed) were regarded as statistically significant. The Z-score indicates how many standard deviations an observation is above or below the mean of the control group. The Vplot that integrates the fold change and P-value is used for depicting the significantly different metabolites. Pathway enrichment analysis used pathway-associated metabolites sets (SMPDB), and pathway analysis bubble plot used mmu set were performed by MetaboAnalyst 4.0 (2) using the online server. Data are presented as mean ± SEM.

**Reference**

1. Goldstein B, Giroir B, Randolph A: International pediatric sepsis consensus conference: definitions for sepsis and organ dysfunction in pediatrics. *Pediatr Crit Care Med* 2005; 6(1):2-8

2. Chong J, Wishart DS, Xia J: Using MetaboAnalyst 4.0 for Comprehensive and Integrative Metabolomics Data Analysis. *Curr Protoc Bioinformatics* 2019; 68(1):e86

**The sequences of primers used for RT-qPCR.**

| Gene | Species | Forward primer (5’-3’) | Reverse primer (5’-3’) |
| --- | --- | --- | --- |
| *Gapdh* | Mice | ACCCAGAAGACTGTGGATGG | CACATTGGGGGTAGGAACAC |
| *Sod1* | Mice | AGCATTCCATCATTGGCCGTA | TTTCCACCTTTGCCCAAGTCA |
| *Sod2* | Mice | GGAGCAAGGTCGCTTACAGA | GTGCTCCCACACGTCAATC |
| *Gpx1* | Mice | TCAGTTCGGACACCAGGAGAA | CTCACCATTCACTTCGCACTT |
| *Cat* | Mice | CCTATTGCCGTTCGATTCTC | CCCACAAGATCCCAGTTACC |
| *iNos*  *Nrf2* | Mice  Mice | GAACTGTAGCACAGCACAGGAAAT  CCGAATTACAGTGTCTTAATACCGA | CGTACCGGATGAGCTGTGAAT  TGAGTAAAAATGGTAATTGCTGTCC |
| *Ho-1* | Mice | GAAGGCTTTAAGCTGGTGATGG | GCATAGACTGGGTTCTGCTTGTT |
| *Srebp1c* | Mice | GGAGCCATGGATTGCACATT | GGCCCGGGAAGTCACTGT |
| *Acly* | Mice | ATCACCGAGGTCTTCAAGGAGGAG | TGGGAATGATGCCGCTGTCAAAG |
| *Fasn* | Mice | CCTGGATAGCATTCCGAACCT | AGCACATCTCGAAGGCTACACA |
| *Fatp1* | Mice | TCTATGACTGCCTGCCACTCTACC | TGCGTGAACTCCTCCCAGATGG |
| *Ppara* | Mice | GCAAGAGAATCCACGAAGCCTACC | TTGTTCATCAAGGAGGACAGCATCG |
| *Cpt1a* | Mice | CTGGATGGCTATGGTCAAGGTCTTC | CAGCAGTATGGCGTGGATGGTG |
