# Supplementary material for: Fibroblast Growth Factor 19 Improves LPS-Induced Lipid Disorder and Organ Injury by Regulating Metabolomic Characteristics in Mice
Source: Oxid Med Cell Longev. 2022 Jul 6;2022:9673512. doi: 10.1155/2022/9673512 (PMC9279090; doi:10.1155/2022/9673512)
Supplement: Supplementary Materials — The Supplementary Material for this article can be found online. [file 9673512.f1.zip › Supplementary Figure 1.docx]

**Supplementary Figure 1. The mRNA levels of genes related to fatty acid metabolism in liver.** Mice were divided into four groups including Control, LPS, only FGF19 treatment (FGF19), and pretreatment with FGF19 followed by LPS administration (FGF19+LPS) (n=6). The mRNA levels of genes involved in fatty acid synthesis (*Srebp1c*, *Acly*, *Fasn*), fatty acid transport (*Fatp1*), and fatty acid β-oxidation (*Ppar**α* and *Cpt1α*). All data are presented as mean ± SEM. ^*^ indicates the significant difference compared with control group, ^&^ indicates the significant difference compared with FGF19 group, ^#^ indicates the significant difference compared with LPS group.
